# ‘Sifting the significance from the data’ - the impact of high-throughput genomic technologies on human genetics and health care

**DOI:** 10.1186/1479-7364-6-11

**Published:** 2012-08-02

**Authors:** Angus J Clarke, David N Cooper, Michael Krawczak, Chris Tyler-Smith, Helen M Wallace, Andrew O M Wilkie, Frances Lucy Raymond, Ruth Chadwick, Nick Craddock, Ros John, John Gallacher, Mathias Chiano

**Affiliations:** 1 Institute of Medical Genetics, School of Medicine, Cardiff University, Cardiff, Wales CF14 4XN, UK; 2 Institute of Medical Informatics and Statistics, Christian-Albrechts University Kiel, Brunswiker Straße 10, Kiel, 24105, Germany; 3 The Wellcome Trust Sanger Institute, Wellcome Trust Genome Campus, Hinxton, Cambridgeshire, CB10 1SA, UK; 4 GeneWatch UK, 60 Lightwood Rd, Buxton, SK17 7BB, UK; 5 Weatherall Institute of Molecular Medicine, John Radcliffe Hospital, University of Oxford, Headington, Oxford, OX3 9DS, UK; 6 Department of Medical Genetics, Cambridge Institute for Medical Research, University of Cambridge, Cambridge, CB2 0XY, UK; 7 Cesagen, 6 Museum Place, Cardiff University, Cardiff, Wales, CF10 3BG, UK; 8 Department of Psychological Medicine, Cardiff University School of Medicine, Heath Park, Cardiff, CF14 4XN, UK; 9 Cardiff School of Biosciences, Cardiff University, Cardiff, Wales, CF10 3AX, UK; 10 Clinical Epidemiology, School of Medicine, Cardiff University, Cardiff, Wales, CF14 4XN, UK; 11 GlaxoSmithKline Medicines Research Centre, Gunnels Wood Road, Stevenage, Hertfordshire, SG1 2NY, UK

## Abstract

This report is of a round-table discussion held in Cardiff in September 2009 for Cesagen, a research centre within the Genomics Network of the UK’s Economic and Social Research Council. The meeting was arranged to explore ideas as to the likely future course of human genomics. The achievements of genomics research were reviewed, and the likely constraints on the pace of future progress were explored. New knowledge is transforming biology and our understanding of evolution and human disease. The difficulties we face now concern the interpretation rather than the generation of new sequence data. Our understanding of gene-environment interaction is held back by our current primitive tools for measuring environmental factors, and in addition, there may be fundamental constraints on what can be known about these complex interactions.

## Introduction

The advent of ‘high throughput’ or ‘next generation’ genomic sequencing technologies has raised expectations of what laboratory genetics has, and will have, to offer to both the clinician and the patient. Our greatly increased ability to generate nucleic acid sequence data raises the question of how this rapidly accumulating mass of new genomic information will be interpreted and when this will become feasible in general clinical practise. How much sense will have been made of the new data within the next 10 to 15 years? What new questions will it be possible for us to pose once these new technologies are readily available to provide the information upon which biologically meaningful answers to these questions can be based? The extended lag time between generating a basic understanding of the pathogenesis of many single gene (Mendelian) disorders and devising effective remedies has long been acknowledged. In the context of complex disorders and the many known quantitative (non-disease) traits, will the lag time between data collection and its interpretation be shortened?

Such questions were addressed at a round-table discussion held in Cardiff in September 2009. This was arranged as part of the work programme of Cesagen, a joint research centre at the Universities of Cardiff (Wales) and Lancaster (England) established by the UK’s Economic and Social Research Council as part of its Genomics Network. Cesagen studies the societal impact of developments in genetics and genomics; the meeting was arranged to explore and discuss ideas as to the likely future course of human genomics. Such technologies will permit researchers to ask new questions of theoretical (biological, e.g. evolutionary) significance and practical (medical and other) application.

The starting point for the discussion was the recent leap in DNA sequencing capability developed by several different commercial enterprises. There was no need to dwell upon the specifics of the technologies; rather, the point was to address the potential benefits for the sciences and clinical medicine of having large volumes of genomic sequence data available, effectively without major constraints of time or cost and alongside an emerging capacity to determine the CpG methylation status of the corresponding nucleotide sequences, the ‘methylome’, and other modifications of chromatin [1]. Several short presentations were made during the round-table sessions, but the emphasis of the meeting was on open discussion among the 20 participants, who have backgrounds in human and evolutionary genetics, clinical medicine, social science and philosophy. The perspectives developed at the meeting have been refined ‘virtually’ since then through multiple cycles of e-mail exchanges.

## Experience of exomics

Useful insights had already been obtained through two large studies: the Genetics of Learning Disability (GOLD) study with complete X chromosomal ‘exome’ sequences from >200 patients with sex-linked cognitive impairment [2] and the description of the full exomes of 12 selected individuals [3]. Frances Lucy Raymond (Cambridge, England) was able to draw a number of general lessons from the GOLD study:

· Sample quality is critical for obtaining reliable DNA sequence data; poor sample quality generates multiple sequence variants per sample, making it difficult to distinguish real sequence from experimental artefact; the assumption that poor quality samples would not amplify was false.

· Truncating sequence variants - termed nulls - are found in approximately 1% of genes on the X chromosome but are nevertheless compatible with normal life in the hemizygous male.

· Missense variants are common, with an average of four unique single nucleotide non-synonymous variants per family with X-linked intellectual disability.

· Data access remains a sensitive issue; there is a need to publish variants and allele frequencies while preserving the anonymity of research participants. This would generally entail publishing aggregate data and new variants rather than individual-specific haplotype information.

These findings emphasise what is already well known, that a disrupted gene need not necessarily lead to a clinically overt disease state, i.e. many genes are dispensable, and even for inactivated essential genes, penetrance will often be incomplete [4,5]. The difficulties inherent in this analysis with regard to the interpretation of previously unreported variants - mutations that may be of pathogenic significance - will be even more substantial when we consider autosomal loci, of which (almost) everyone will have two copies [6]. Additional new approaches to determine the functional significance of genomic variation will be required if any coherent interpretation of the approaching deluge of data is to be feasible [7].

## Epigenetics

Epigenetic influences together introduce an additional layer of complexity over and above the functionality of genomic DNA sequences [4]. The inter-relationship of DNA sequence and DNA sequence modification has not yet been adequately addressed in either theory or experiment; it should, however, be noted that this relationship is likely to be bi-directional with certain polymorphic variants influencing methylation status and the methylation status in turn influencing mutability. Ros John (Cardiff, Wales) introduced this topic and its relationship to known imprinting phenomena, referring to both epidemiological and experimental work, *viz.* records of the Dutch famine at the end of World War II [8] and contemporary work with the agouti mouse [9]. The term ‘epigenetics’ includes the study of both conventional gene-gene (GxG) interactions, long recognised in principle but in practise exceedingly difficult to measure in humans, and the still under-researched area of ‘predictive adaptive responses’. The latter has been elaborated theoretically [10], triggering very interesting - but so far perhaps more tantalising than illuminating - laboratory work on the possible influence of early life experiences (before or soon after birth) on patterns of DNA methylation and subsequent disease susceptibility in the face of dietary challenges and other environmental circumstances.

Our understanding of the intricate role of the epigenome in directing differentiation, communicating cell fate and mediating adaptive response will be informed by comprehensive surveys of epigenetic marks across space (cell type/state) and time (development and ageing), and in response to environmental challenges (diet, stress). To this end, a number of ingenious genome-wide, high-throughput technologies have been developed. Some are based on the ability to selectively capture methylated fragments of the genome using methyl-binding proteins and then sequencing these fragments. MeDIP-seq, MBD-seq and MethylCap-seq have all been applied successfully, although with differences in selectivity for specific sequences [11,12]. A readout of the whole genome methylation status can also be obtained by applying a bisulfite-sequencing technique, whereby genomic DNA is exposed to HSO_3_^−^, which deaminates only the unmethylated (and, hence, unprotected) cytosine residues resulting in a change in the DNA sequence. This technique may provide greater coverage of the genome [13]. The genome-wide assessment of histone modifications is a more complex task since these modifications are numerous, and activating and silencing marks are not necessarily mutually exclusive [14]. Chromatin immunoprecipitation followed by high-throughput sequencing is the preferred technique for determining the genome-wide locations of specific histone modifications [15,16]. Although there are still issues, in particular, the biassed representation of certain sequences, computational analyses and further technical refinements will undoubtedly increase the sensitivity of these techniques and further reduce the costs.

One potentially very important aspect of GxG and gene-environment (GxE) interactions that may limit their importance, or at least our ability to detect them, is the small effective population size (N_e_ in population genetic notation) of humans [17]. Another important question is the extent to which the assessment of CpG methylation can serve as an adequate biomarker for the full range of epigenetic influences. A systems biology approach to the recognition of biological networks may be one way of addressing GxG and GxE interactions in humans [18,19], given that the types of breeding experiments pioneered in *Drosophila* species are clearly ruled out in humans, not only on ethical grounds but also by the restrictions of timescale available to researchers. The collection of biological samples and data on the large Helmholtz cohort in Germany is intended to address some of these issues.

## Behaviour and psychiatry

When one turns to consider human behaviour and psychiatric disorders, rather than the (relatively) simple phenotypes associated with human growth or with metabolic and developmental disorders, additional layers of complexity become evident in the GxE interactions. As Anita Thapar (Cardiff, Wales) made clear, there is a pressing need for good measures of both the environmental factors and the behavioural phenotypes of potential interest. The GxE interactions can be looked at as purely statistical phenomena or, more interestingly and perhaps more importantly, as phenomena of potential biological meaning. Here more than anywhere, and in deference to the great David Hume, it is essential to avoid the all-too-easy slide from the observation of an association into unwarranted assumptions about causation. Thus, if one observed an association between attention deficit hyperactivity disorder in children and maternal cigarette smoking, what causal influences might have been at work, - e.g. an environmental effect of smoking in pregnancy or a common genetic basis to both behaviours? One opportunity to clarify such questions, although difficult to establish, would be longitudinal studies of children born following conception by IVF. Some of these questions are profound, touching our very humanity; for instance, how (through what process) does the early maltreatment of children result in later antisocial behaviour? How may social interventions be designed effectively to interrupt such pathogenetic linkages, for the long-term benefit of society as a whole? The Bradford-Hill criteria [20] address this problem of causation but have yet to be fully tested in the genomic era.

Improved methods of measuring environmental factors and their behavioural correlates are clearly required for the full benefits of the progress in genome sequencing to be attained; these measurements should be made without lapsing into implicitly deterministic assumptions.

## Common disease genetics

Michael Krawczak (Kiel, Germany) challenged the assumptions underlying much ‘common disease genetics’ - especially the limitations of its most influential models. He expanded upon three critical underlying assumptions made by Reich and Lander [21,22]:

· The allelic spectrum of common disease genes is simple.

· There was a single, sudden expansion event of the human population (at least, the out-of-Africa population) from 10,000 members to its modern size.

· The part played by selection in the spread of alleles predisposing to common diseases was relatively small.

This model has led to the neglect of new mutants arising from existing variants during the turnover of the allelic spectrum of common disease. Krawczak explained why these assumptions are highly implausible and what effect this is likely to have had on the current allelic spectrum of genetic predisposition to complex disease. In particular, as is apparent both when other modelling approaches, such as coalescence theory, are used and from the data currently accumulating:

· The allelic spectrum of complex disorders, like that of Mendelian disorders, is likely to be diverse.

· There may be only a weak correlation between the risk of disease and the population frequency of risk alleles.

· The functional effects of predisposing alleles may be weakly or even inversely related to the associated disease risk (some predisposing alleles may even, counter-intuitively and due to stochastic processes, be more frequent in unaffected than affected individuals).

· The mutational load in unaffected individuals may be particularly high for common diseases.

## Mapping the ‘polygenes’

Krawczak also discussed the limitations of the available strategies for mapping the loci which contribute to the common, complex disorders. The power of the affected sib pair (ASP) linkage approach has been regarded as low [21] in comparison to association studies employing the transmission disequilibrium test (TDT), not a case–control design, but that view also had shortcomings:

· The paper assumed knowledge of the risk allele when assessing the TDT but not when assessing the ASP method.

· The relative risks invoked were unrealistically high.

· The association approach was found to be advantageous for studies of candidate genes (or regions) but not necessarily for genome-wide methods exploiting linkage disequilibrium, such as genome-wide association studies (GWAS).

There are good grounds for using both family studies and GWAS to utilise all the available evidence [23]. Family-based approaches that could be used with whole-genome sequencing (potential scenarios) include:

· sequencing the index patients and evaluating the inheritance pattern of ‘interesting’ genes or gene regions in their families

· sequencing the parents of the patient, comparing the results to population controls, and using TDT on ‘promising’ alleles

· assessing the co-inheritance pattern of unlinked genes in families to derive candidate regions for GxG interactions

· defining and assessing pre-disease or sub-clinical disease states in genetically-defined high-risk individuals identified in families.

Andrew O M Wilkie (Oxford, England) mentioned that the supposed ‘triumphs’ of the common disease/common variants (CD/CV) model conveniently ignore the fact that the relative risks conferred by susceptibility alleles cluster much more closely around 1.0 than anyone envisaged two decades ago; relative risks above 2.0 are decidedly unusual. Hence, the majority of heritability remains unexplained, and useful personalised prediction in healthy individuals (largely based on family history) has hardly improved over this time.

Additional challenges to our understanding include the greater heritability of diseases (e.g. schizophrenia, as presented by Nick Craddock of Cardiff, Wales) than would be expected given the CD/CV model of disease causation and the greater difficulty of interpreting any estimates of heritability when the disease phenotype results from the joint action of several predisposing genes (as Helen M Wallace (Buxton, UK) reminded us). Whether these insights into pathogenesis will eventually convert to promised improvements in human health remains to be seen.

There was discussion about the possible ‘replication’ of the results of GWAS, led by Mathias Chiano (Stevenage, England). Even the question of what exactly ‘replication’ would entail needs to be clarified, as when specifying the population(s) from which study participants are drawn. If an attempt is made to replicate a given study in a different population, the meaning of a negative result will be unclear because the relevant genetic factors may differ between this and the original population; there are no easy answers in relation to population differences.

## Selection

Chris Tyler-Smith (Hinxton, England) continued with the theme of human history and the contemporary traces of past selection. Selection can take many forms but the presence of high levels of polymorphism suggests either an ancient origin for neutral mutations or the maintenance of polymorphism by fluctuating or balancing selection. Recognising past selection is possible by (i) counting offspring, (ii) looking for patterns of DNA variation in populations that depart from neutrality (such as long haplotypes, skewed allele frequency spectra, large differences between populations), and (iii) functional studies. Recent genomic studies comparing populations have drawn upon the second category of evidence that detects the net effects of positive selection over millennia, but these have mostly been based on simple models of selection and are less able to detect phenomena such as heterozygote advantage, frequency-dependent selection, disruptive selection - selection for both homozygotes - or antagonistic but stabilising selection with contrary effects at different stages of the life cycle in the two sexes or in different environments.

While there are grounds for thinking that selective ‘sweeps’ have been uncommon in recent human evolution [24], the role of selection in maintaining substantial levels of polymorphism remains less clear. Although a biological (natural selection-based) account for many genetic observations consistent with selection remains elusive, this could be the result of the challenging nature of such studies. The persistence of polymorphism without a clear genomic signature may result from changes in the strength and direction of selection. Despite these complexities, there are widely accepted examples of positive selection, including disease-associated variants and those influencing visible traits such as skin colour or hair, where sexual selection through gene-culture interaction may have been relevant [25,26].

## Human genetic variation: the 1000 Genomes Project

Results from the 1000 Genomes Project [27] and other studies indicate that, despite the low level of genetic variation in humans compared with other apes, there are still enormous inter-individual differences in genome sequence, which can now be identified in a comprehensive way at the level of CNVs, indels and SNPs. Notably, there are on average 250 to 300 loss-of-function variants such as ‘nonsense’ or splicing SNPs in the genome of the average ‘healthy’ person, 50 to 100 previously implicated, disease-associated variants and approximately 80 genes that vary in copy number. As suggested by the GOLD study (*op cit*), this greatly complicates the clinical interpretation of an individual's genome sequence.

The first goal of the 1000 Genomes Project has been to capture important data about background human genetic variation which will be essential in studies of both inherited and acquired disease. The samples being studied:

· were initially drawn from urban populations in four countries (HapMap Nigerian, Japanese, Chinese, and families with European ancestry from the USA) but additional samples will be drawn from elsewhere, including South Asia

· were from anonymous individuals with no phenotypic information provided (except sex, population of origin and that they are adults competent to provide consent), but do have consent for free web release of the sequence data, and are available as cell-lines to researchers

· because full × 30-depth sequencing to detect heterozygous variants in a single individual effectively would be too costly, low coverage sequencing of samples from many individuals is being used to capture the variants shared between individuals; the full project aims to detect essentially all polymorphic variants (i.e. those at >1% frequency) in the geographical areas investigated that lie in the accessible part of the genome.

### 1000 Genomes pilot studies

· Samples (179) at depth *×* 2-4 should identify most variants present at >5% - so far, 17.2 million SNPs have been found, of which >50% were novel.

· Two trios sequenced at × 30-60 revealed many ‘new’ (*de novo*) mutation events (see below)

· Approximately 1,000 genes were sequenced deeply in samples from 700 volunteers.

The next task: it will be necessary to draw upon large population studies to seek evidence of variable mutation penetrance.

## Mosaicism and foetal DNA in the maternal circulation

High-throughput sequencing is also opening up the detection of mosaicism, which is likely to have many biological consequences in addition to neoplasia [28]. With a read depth of × 30, it is difficult to detect mosaicism at or below the level of 5% because of the 1% error in typing each nucleotide position. This could have applications in the study of cancers, e.g. the evolution of individual tumours as reflected in tumour-specific DNA [29], and in prenatal diagnosis, where free foetal DNA is currently used to look for alleles not present in the mother, such as Rhesus or Y chromosome sequences, but is likely soon to be applicable to population screening, especially for trisomy 21 [30-32]. Greater read depths will be required for clinically important applications reliant upon the detection of low levels of mosaicism [33].

## Phenotypes and taxonomy

Craddock led a discussion on disease phenotypes and their relation to disease taxonomy, especially in relation to psychiatric disorders. The definition of a phenotype is crucial as it may be influenced by numerous factors including ‘culture’ and tradition, established or desired patterns of health service utilisation, interacting environmental variables and previously accepted models of disease causation.

There has been a growing awareness that the specific single genes in which mutations cause a few rare disorders may also be (more loosely) associated with disease predispositions apparent in GWAS. Thus, rare mutations in the *CACNA1C* gene can result in a multisystem disorder, manifesting as cardiac dysrhythmias, epilepsy, autism, cognitive impairment and abnormal physical features, whereas variation in the same gene is more weakly associated with bipolar disease, schizophrenia and unipolar depression, as evidenced by GWAS. The benefits of identifying specific genes in which variation contributes to disease susceptibility include understanding the pathogenesis, establishing a diagnosis, devising new therapies and selecting the most appropriate treatment for the individual patient.

Following the course of human genetics research over the past 15 years, one may perceive a shift in focus from rare to common diseases and from genes of large effect in which mutations cause disease to genes of small effect in which variants merely modify the risk of disease. Mutations in genes of large effect are usually accepted unequivocally as important causal factors; the modifying genes of lesser effect can, however, also provide insights into pathogenesis when several loci in the same developmental or functional pathway interact to contribute to a specific disease. It is also clear that defining the phenotype is, itself, an iterative process, taking the researcher back and forth between the phenotype and the genotype. The researcher can use different values of a given parameter in order to establish which yields the clearest discrimination between individuals with and without disease.

The finding that sequence variation at some of the same loci influence both schizophrenia and bipolar disease has cast doubt on the validity of the Kraepelinian dichotomy of the psychoses into these categories [34,35]. The exclusion of ‘intermediate’ categories of patients from much previous research had in effect prevented this dogma from being challenged in the past - it became a closed ‘view of the world’ (a prophecy that was both dogmatic and self-fulfilling).

More recently still, it has become clear that new mutations at many loci contribute very substantially to schizophrenia, confirming the indications from studies of *de novo* CNVs that *de novo* rare mutations contribute importantly to this relatively common disease [36,37] and demonstrating that the reservations of Krawczak and others about the CD/CV model were warranted.

## Mutation and selection at single loci

There is still much to be learned from the detailed study of single loci, in addition to genome-wide research that does not assume the applicability of the CD/CV model of complex disorders. As an example, Wilkie presented information from his long term studies of the fibroblast growth factor receptor (*FGFR*) genes.

As background to the *FGFR* studies, germline mutation rates have been estimated to be about 2.5 × 10^−8^[38] or 1.8 × 10^−8^[39] - equivalent to approximately 120 nucleotide substitutions per birth. The fitness of amino acid substitutions has been estimated [40,41], and purifying selection is thought to act on 2.5% to 5% of the genome (of which 1% will be coding sequence). This will lead to 1 to 3 harmful amino acid substitutions per birth, plus a similar number from each previous generation. Recent data from the 1000 Genomes Project suggest a slightly lower rate of new mutations, closer to 10^−8^ per base pair per generation, and that the influence of purifying selection can be detected through a reduction in genetic variation at distances of up to 100 kb from genes, implying that few parts of the genome are entirely unaffected by such selection. Similarly low rates of mutation have been found by others [42].

Wilkie described how he set out to explain the apparently very high levels of mutation in specific genes using the *FGFR* genes as a model. The most frequent transition mutation in the human germline is the *FGFR3* mutation that causes achondroplasia, but Wilkie chose to study the Apert syndrome mutation 755 C > G in *FGFR2*, which is the most common transversion mutation in humans. The mutations originate exclusively from the healthy fathers of the affected individuals, who tend to be older than average for the population (paternal age effect). From the analysis of normal sperm and testes, it can be deduced that the positions that mutate in *FGFR* genes are not true mutation hotspots; rather, there is positive selection for spermatogonia carrying the mutation because the mutation confers gain-of-function properties upon the encoded protein [43-45]. Wilkie looked for these mutations in an uncommon testicular tumour - spermatocytic seminomas - and a comparable spectrum of mutations was found here as in congenital disorders and in bladder tumours: they all act on the Ras/MAPK pathway, which plays a central role in regulating proliferation and other critical cellular processes. The testis may be viewed as a ‘bioreactor’ for selfish mutations that promote the clonal growth of spermatogonia harbouring one of these growth-promoting mutations [46]. The effects of each mutation vary with the degree to which it is activating:

1) highly activating mutations: lethal disorders or testicular tumours

2) moderately activating mutations: congenital malformation syndromes exhibiting a paternal age effect

3) weakly activating mutations: rare sequence variants that may predispose to a wide spectrum of disorders, involving for example neurodevelopment and cancer predisposition

It should be noted that the latter category of mutations will be undetectable by GWAS, and so may contribute to missing heritability [47].

The paternal age effect for schizophrenia may occur through a similar mechanism. It should be remembered that more than 1% individuals with schizophrenia have a *de novo* CNV, often a rare but recurrent CNV [48], while CNVs have been shown to be much more common in cases of schizophrenia than in controls (5% of controls, 15% to 20% of cases) [49] and, interestingly, to overlap with the CNVs found in attention deficit hyperactivity disorder [50]. However, the frequency of *de novo* CNVs is high enough in controls that such *de novo* events cannot be taken as *ipse facto* proof of pathogenicity.

## Pharmacogenetics

Chiano led a discussion on pharmacogenetics and medical practice. Genetics is just one of the factors that influence the safety and efficacy of particular drugs. These factors include dosage, ‘the environment’, compliance, other drugs, diet, age and the co-occurrence of other diseases. Efficacy can vary from 80% for COX-2 inhibitors to 25% for cancer chemotherapy. The abacavir-associated hypersensitivity reaction can occur in 5% to 8%; there is a major susceptibility locus in the HLA region [51,52]. Proponents have been disappointed at the slow pace of introduction of pharmacogenetic testing into clinical practice. Meanwhile, somatic genetic tests are proving to be helpful in guiding the treatment of some malignancies but have been slower to enter regular clinical practice for a wider set of indications despite the longstanding recognition of the relevant pharmacogenetic phenomena: additional criteria of utility and cost-effectiveness also need to be fulfilled [53].

## Privacy and consent

Ruth Chadwick (Cardiff, Wales) led a discussion on issues of privacy and consent in genomic research. It is becoming impossible for medical researchers to guarantee privacy to the research participants they recruit - especially with the pressure from funding agencies who insist upon open-access archiving of genomic sequence data, as these data inevitably contain potentially identifying information. Indeed, it would now be misleading to promise privacy of personal genome information to research participants in exchange for consent to donate samples.

There are difficulties at the level of the technology (sample collection and storage, and data generation, storage and access) and the interpretation of information collected. It has been argued that the very concept of privacy will have to be renegotiated in the context of the rationale(s) for data sharing, especially at international level. Indeed, there has been a move from ‘traditional’ notions of informed consent to one of a broad consent and then to open consent. One must either strengthen the traditional practises of consent - ensuring that participants understand the difficulties of ensuring privacy before giving consent (while at the same time pursuing the data protection strategies that are possible) - or re-think the whole notion of privacy [54]. In clinical practice, there may be recurrent difficulties when molecular studies of a malignancy, designed to guide therapeutics, thereby, also yield unwelcome information about prognosis or implications for close relatives [55]. In the context of research, however, new forms of consent (e.g. broad consent taken online) may well be readily accepted [56]. One approach is for the researcher to move from guaranteeing (and so protecting) privacy to practising veracity, explaining to potential participants that their data will be accessed by, and shared with, others; those consenting to research have to be open to this [57].

Those participating in ‘personal genomics’ would be deluding themselves if they thought that privacy was still possible once their genome sequences became accessible online [58,59]). This is the age of bioinformatics, surveillance, Facebook and Twitter. There is inevitably a risk of personal consequences for research participants when their data are released. These will take different forms under different circumstances. Access to health care may become more difficult in countries without state health care or universal and compulsory health insurance, as when health care is made available through commercial insurance schemes. In addition, if information about other family members becomes available, then biological family relationships might be shown to differ from the pattern of social relationships presented in public (e.g. paternity may have been misattributed).

One perspective reveals a clash of research cultures: molecular scientists (who seek instant open access to all data to maximise the scientific - and perhaps commercial - exploitation of the data) and the clinicians (who are often more insistent upon protecting their patients and more modest in their assessment of the likely short-term benefits to the participants, and other patients, of the research itself).

One key notion is that of the acceptable uses for research data. Participants tend to trust ‘the system’ to use their data in a ‘good’ way. The system of research governance is designed (in part) to ensure that such trust is warranted - although the system of governance also serves other, more institutional purposes. The prospect of internet-based marketing corporations using access to research data and to electronic health records as an opportunity to market more products seems both manipulative and cynical.

The concept of ‘consent’ is also problematic in ways unrelated to the concept of privacy. As Raymond explained, families participating in research studies could allow their genuine altruism to lead them to sign the research consent forms because their thoroughly worthy impulses blinded them to the potential for problems to arise from participation. Such problems might include results of uncertain significance, which may remain difficult to interpret for many years or whose significance might only be clarified through the performance of family studies that could be confusing or distressing; even with such family studies, the significance of the results may remain opaque. If the reasons underlying the importance and relevance of consent to research were explained, then many families might decline participation.

One response to these possibilities has been the suggestion that a solution should be found through information technology itself, with the development of a system of ‘data enclaves’, within which researchers can utilise data in a predefined analysis but from which they cannot export any data about individuals - only aggregate data and the derived results of analysis.

## Limited clinical utility of genetic association

Wallace set out her answer to the important question, ‘What are the (biological) limits to the predictive value and clinical utility of the ‘predict and prevent’ strategy?’ An early approach to this led to the conclusion that environmental interventions targeted by genotype might significantly reduce the incidence of some diseases [60], dependent on the magnitude of gene-environment interactions (in the statistical sense of their influence on risk at a population level). However, there was an error in the measure of clinical utility used in this paper, and in addition, it took no account of the proportion of the population in the high-risk group (thus, erroneously suggesting that a genetic test which identified the whole population as at high risk would have high clinical utility). Further, the aetiology of a given disease will place constraints on the potential magnitude of both the genetic component of a disease and of any gene-environment interaction. Wallace set out her approach to assessing clinical utility in the context of genetic testing [61].

Utility, U_GE_ = population impact − gamma, where gamma is the fraction of the population classified as at high genotypic risk, and the population impact is the avoidable proportion of disease occurring in this fraction of the population, compared to the population as a whole.

Utility is larger if gamma is small, and targeting is only effective in the presence of GxE interactions. If there is no GxE interaction, genotyping performs no better than random selection in terms of reducing the incidence of the disease in a population. If high-risk individuals have less to gain from intervention than the low-risk group, then targeting has negative utility. There is a range of solutions for any given disease, which can be mapped out if data on relative risks from both twin and family studies, plus environmental data, is known. Each solution depends on the model that is assumed for gene-gene and gene-environment interactions. Because of the number of alternative models, the sample sizes required to evaluate or validate risk predictions may be too high, and it could be impossibly difficult to generate good association (GWAS) data and integrate it with environmental information [62]. The model also confirms earlier findings that heritability estimates can be high even in the complete absence of any genetic component to a disease [63] and that gene-gene and gene-environment interactions, as well as the equal environments assumption, may account for some (or even all) of the ‘missing heritability’ of complex diseases.

It should be noted that recent modelling of heritability and GWAS data suggest that a much greater fraction of the heritability of quantitative traits can be accounted for by GWAS data than had been found previously, once allowance is made for the incomplete linkage disequilibrium between the genotyped SNPs and the ‘true’ causal factors [64-66]. However, such modelling is still unable to identify the factors involved and cannot deliver improved capacity to predict disease. Furthermore, only a small proportion of the calculated heritability has yet been identified for any common disease [67].

## Conclusion

The space provided by this workshop and the subsequent e-mail exchanges has served to clarify our thoughts and allowed several modest conclusions to emerge, although unanimity was not achieved on all issues. Four points, in particular, became progressively clearer during these discussions.

First, the new insights derived from the application of high throughput sequencing are likely to transform our understanding of human biology, especially in the context of human genetic disease and evolution. Clinical applications of human genome sequencing are emerging in oncology and reproductive genetics and are beginning to impact upon other clinical areas and disciplines. The old debates about nature vs. nurture are likely to re-emerge in new guises, and great care will be needed in case fruitless debate consumes too much energy or misconceived arguments lead to the inappropriate application of theory in policy and practise across various walks of life, most especially psychiatry, education and the law.

Second, and despite a full acceptance of this first point, major problems remain in moving from the rapidly accumulating raw sequence data and catalogues of genetic variation to warranted conclusions in their interpretation. These problems include questions relating to the clinical utility of conclusions drawn on the basis of genetic association studies that lack a plausible pathogenic mechanism underlying the observed association. There will inevitably be uncertainties in the face of a morass of interacting variables, but some of the difficulties should be resolved over time - although perhaps on a timescale of decades rather than months. In addition, our current measures of environmental factors are primitive, and our ability to record the variation in such factors over a lifetime of many years is still crude.

Finally, and despite the continuing advances in information technologies, the accumulation of both genomic and environmental data may outstrip the capacity of our information systems to store and analyse the data [68]. These limits to computability may not be merely practical in nature - to be overcome once technology has moved on - but may represent constraints as to what can be known or calculated even in principle, given limits to global population size, the nonrandom distribution of genotypes and the exponentially increasing number of statistical comparisons that can be made; how can one correct adequately for such astronomical numbers of comparisons? Deductive, hypothesis-driven research on the basis of such data will be challenging!

Acquiring the necessary genome sequence data appears now to be almost the easy step by comparison with the quantification of environmental factors and of human phenotypes, the physical limits to data storage and constraints in principle to computability and interpretation.

## Competing interests

The authors declare that they have no competing interests.

## Authors’ contributions

AC initiated the project and led the writing of this report. RC ensured the fit between the project and the scope of Cesagen/ESRC support. All authors participated in the discussion and the development of the report into this article and then read and approved the final manuscript.
